# Selected reaction monitoring assays in mesenchymal stem cells from osteoarthritis patients

**DOI:** 10.1186/1559-0275-11-33

**Published:** 2014-09-01

**Authors:** Emilio Camafeita, José-Ramón Lamas, Enrique Calvo, Pilar Tornero-Esteban, Juan-Antonio López, Benjamín Fernández-Gutiérrez

**Affiliations:** 1 Unidad de Proteómica, Centro Nacional de Investigaciones Cardiovasculares, Madrid, Spain; 2 Rheumatology Service, Hospital Clínico San Carlos, Profesor Martín Lagos s/n, Madrid E-28040, Spain

**Keywords:** Osteoarthritis, Mesenchymal stem cells, Selected reaction monitoring

## Abstract

Osteoarthritis (OA) is considered the most prevalent form of arthritis. The aim of this study was to verify potential protein OA biomarkers by applying Selected Reaction Monitoring (SRM) assays to protein extracts obtained from Bone Marrow-Mesenchymal Stem Cells (BM-MSCs) isolated from OA patients.

BM aspirates were obtained from the femoral channel of OA patients at the time of surgery and from the femoral channel of hip fracture subjects without OA during hip joint replacement surgery for the treatment of subcapital fracture.

SRM results verified the differential expression of several protein biomarkers in BM-MSCs from OA patients.

## 

Proteomic approaches have proposed numerous protein factors potentially involved in pathological processes including osteoarthritis (OA) [1], the most prevalent rheumatic disease [2]. While the validation of such potential biomarkers has traditionally relied on immunoassays, specific antibodies may not be available [3], and selected reaction monitoring (SRM) has recently emerged as a promising application in medical screening due to its ability for multiplexed, high-throughput analysis as well as its sensitivity and quantitation capacity [4,5]. In a previous work we have described the regulation of several proteins in bone marrow mesenchymal stem cells (BM-MSCs) from OA patients [6]. The potential application of MSCs in cell therapies aimed at rheumatic diseases has aroused great interest [7], and our results suggested the preactivation of these cells by signaling events produced by the subchondral bone. To verify these potential protein OA biomarkers, SRM assays have now been applied to protein extracts from BM-MSCs isolated from OA patients.

BM aspirates were obtained from two OA patients (age: 52 and 89 years) and three hip fracture subjects without OA (mean age 73.3 years, range 47–90) during hip joint replacement surgery. Hip fracture subjects without OA had a densitometric T-score > 2.5 SD (Hologic QDR-4500C) performed after surgery. All samples were obtained after patients gave their informed consent, and this study was approved by the local institutional ethics committee. After culturing the cell-containing fraction of BM aspirates as previously described [6], five pellets of approximately 2 × 10^6^ confluent cells at the third passage were obtained. Then the cells were lysed, protein concentration was measured as previously described [6] and the five resulting protein extracts (110 μg each) were separated by SDS-PAGE on a 12% polyacrylamide gel. The proteins were stained and digested with trypsin as described in [6].

Since peptide precursor and fragment data from our previous, DIGE-based experiments [6] were generated using MALDI ionization, additional data based on ESI were necessary to choose the most appropriate transition pairs. For that, prior to SRM half the sample amount (55 μg) was subject to shotgun analysis on an LTQ-Orbitrap XL ETD (Thermo-Fisher) ESI mass spectrometer as described previously [8]. The precursor-diagnostic transitions to be assayed by SRM were chosen by carefully examining the MS/MS spectra corresponding to the peptides most reliably identified in the shotgun analysis (data not shown). To keep the transition list at a size that would not compromise the performance of the mass spectrometer, two to three transition pairs were chosen for eight out of the 38 proteins previously found regulated in the MSCs from OA patients [6]. A 4000 Q-Trap (AB Sciex) hybrid instrument was used for the multiplexed, non-time-scheduled SRM analysis of the above selected pairs followed by enhanced resolution scans (for charge and mass determination) and enhanced product ion scans (for induced fragmentation) along a single chromatographic run (80 min) per sample (55 μg). The analysis of mass spectra was carried out using the Analyst 1.5 software (AB Sciex). Relative peptide quantification was performed by measuring the area of the extracted ion chromatogram (XIC) peaks from selected precursor ions. Two transitions from pyruvate kinase peptides that showed comparable spectral counts across the two OA and the three control samples in the shotgun analysis were used to normalize XIC areas and to check that retention time variability was within ±2.5 min. In addition, the presence of the MS/MS fragmentation spectra from the corresponding precursor ions was manually checked along the XIC peaks to rule out interference from unrelated ions. The statistical comparison of the OA and control groups was performed based on *parametric Student’s t test and/or non-parametric Mann–Whitney U test.* Values with p < 0.05 were considered significant.

SRM results enabled the verification of a five-protein set for which significant differences were measured between patients and controls (Table 1 and Figure 1). The ratios calculated on the basis of the transition areas shown in the corresponding XIC and box and whisker plots (Figure 1) were comparable to those obtained by DIGE (Table 1). Limitations of this study have arisen from the reduced number of samples and cells available; thus, time-scheduled assays would have enabled SRM analysis of a much higher number of transition pairs, therefore expanding verification to other proteins. The availability of higher sample amounts would also enable: i) The analysis of technical replicates to improve statistical power; ii) The use of retention time standards or reference synthetic peptides to control chromatographic reproducibility; and iii) To minimize the use of precursor peptides with sub-optimal proteotypic nature by increasing sequence coverage in shotgun experiments

**Table 1:** Precursor-fragment transitions significantly different between OA and control patients

**Protein name**	**Precursor sequence**	**Q1(charge)**^**a**^	**Q3(ion)**^**b**^	**XIC**^**c**^	**DIGE**^**d,e**^		**SRM**^**e**^	
					**Ratio p-value**		**Ratio p-value**	
Tubulin α6	DVNAAIATIK	508.3(+2)	801.4(y_8_)	a	−1,76	0.007	−2.17	0.032
	LISQIVSSITASLR	744.4(+2)	933.5(y_9_)	b				0.017
Cathepsin B chain B	LPASFDAR	438.7(+2)	666.3(y_6_)	c	+1.90	0.036	+4.21	0.005
	NGPVEGAFSVYSDFLLYK	1003.5(+2)	1048.5(y_8_)	d				0.031
Dihydropyrimidinase-like 2 variant	VFNLYPR	454.7(+2)	662.3(y_5_)	e	−2.52	0.028	−4.25	0.006
	IAVGSDADLVIWDPDSVK	950.5(+2)	545.7(y_6_)	f				0.033
	IAVGSDADLVIWDPDSVK	950.5(+2)	846.4(y_7_)	g				0.008
L-caldesmon II	GNVFSSPTAAGTPNK	724.4(+2)	856.5(y_9_)	h	−1,93	0.008	−2.89	0.009
Destrin isoform B	YALYDASFETK	654.3(+2)	797.4(y_7_)	i	+4.30	0.006	+4.02	0.025

^a^Peptide m/z value filtered in the fisrt quadrupole; ^b^Fragment m/z value filtered in the third quadrupole; ^c^As depicted in Figure 1; ^d^From the comparative analysis of differential protein expression [6]; ^e^The symbols ‘ + ’ and ‘-’ indicate increased or decreased abundance in OA samples relative to controls, respectively; DIGE, differential in-gel electrophoresis; m/z, mass-to-charge ratio; OA, osteoarthritis; SRM, selected reaction monitoring; XIC, extracted ion chromatogram.

**Figure 1:**
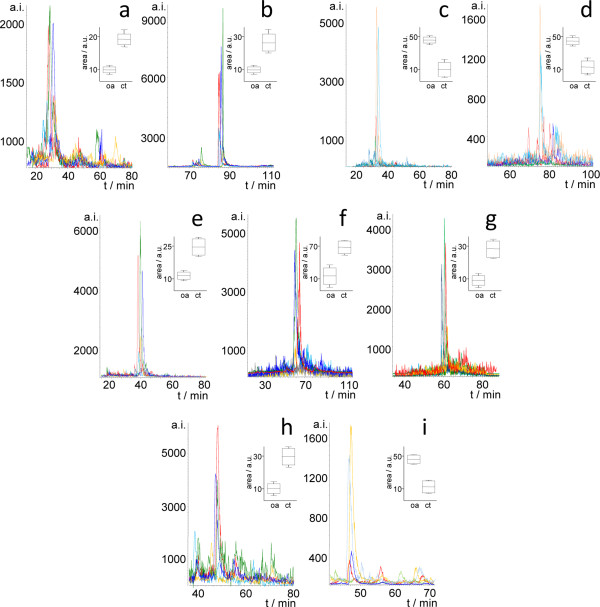
**SRM assays with proteins previously found regulated in MSCs from osteoarthritic patients.** For the measurements which produced significant differences (p < 0.05) between patients () and controls () the extracted ion chromatogram (XIC) is shown together with maximum, minimum and average ± SD values obtained by measuring peak areas corresponding to the transitions detailed in Table 1**(a-i)**. Two transitions from pyruvate kinase peptides (GDLGIEIPAEK: 571.3(+2)/686.4(y6); IYVDDGLISLQVK: 731.9(+2)/574.4(y5)) were used to normalize the data. ct, controls; MSCs, mesenchymal stem cells; oa, osteoarthritis; SD, standard deviation.

Based on SRM we have verified the differential expression of potential protein biomarkers in BM-MSCs from OA patients. Results reinforce the hypothesized preactivation of these cells by signaling events produced by the subchondral bone and corroborate the feasibility of using SRM for the quantitation of biomarker sets in clinically relevant samples.

## Abbreviations

BM: Bone marrow; DIGE: Differential in-gel electrophoresis; ESI: Electrospray ionization; MALDI: Matrix-assisted laser desorption/ionization; MSC: Mesenchymal stem cell; MS/MS: Tandem mass spectrometry; OA: Osteoarthritis; SDS-PAGE: Sodium dodecylsulfate polyacrylamide gel electrophoresis; SRM: Selected reaction monitoring; XIC: Extracted ion chromatogram.

## Competing interests

The authors have no conflicts of interest to declare.

CNIC is supported by the Ministerio de Economía y Competitividad and the Fundación Pro CNIC. Support was also provided by FIS PI10/00178 and RETICS Program, RD08/0075 (RIER) from Instituto de Salud Carlos III (ISCIII). JRL is a recipient of Miguel Servet Program from Instituto de Salud Carlos III (ISCIII).

## Authors’ contribution

EmC: SRM performing, writing of the manuscript and discussion of results. JRL: obtaining of mesenchymal stem cells and discussion of results. EC: SRM performing and discussion of results. PTE: obtaining of mesenchymal stem cells and discussion of results. JAL: SRM performing and discussion of results. BFG: selection of patients, writing of the manuscript and discussion of results. All authors read and approved the final manuscript.
